# IgG_1_ pan-neurofascin antibodies identify a severe yet treatable neuropathy with a high mortality

**DOI:** 10.1136/jnnp-2021-326343

**Published:** 2021-08-16

**Authors:** Janev Fehmi, Alexander J Davies, Jon Walters, Timothy Lavin, Ryan Keh, Alexander M Rossor, Tudor Munteanu, Norman Delanty, Rhys Roberts, Dirk Bäumer, Graham Lennox, Simon Rinaldi

**Affiliations:** 1 Nuffield Department of Clinical Neurosciences, University of Oxford, Oxford, UK; 2 Department of Neurology, Morriston Hospital, Swansea, UK; 3 Manchester Centre for Clinical Neuroscience, Salford Royal NHS Foundation Trust, Salford, UK; 4 MRC Centre for Neuromuscular Disease, National Hospital for Neurology and Neurosurgery, London, UK; 5 Department of Neurology, Beaumont Hospital, Dublin, Ireland; 6 Department of Clinical Neurosciences, Cambridge Institute for Medical Research, Cambridge, UK; 7 Department of Neurology, Great Western Hospital, Swindon, UK; 8 Department of Neurology, Oxford University Hospitals NHS Foundation Trust, Oxford, UK

**Keywords:** neuropathy, neuroimmunology

## Abstract

**Objectives:**

We aimed to define the clinical and serological characteristics of pan-neurofascin antibody-positive patients.

**Methods:**

We tested serum from patients with suspected immune-mediated neuropathies for antibodies directed against nodal/paranodal protein antigens using a live cell-based assay and solid-phase platform. The clinical and serological characteristics of antibody-positive and seronegative patients were then compared. Sera positive for pan-neurofascin were also tested against live myelinated human stem cell-derived sensory neurons for antibody binding.

**Results:**

Eight patients with IgG_1_-subclass antibodies directed against both isoforms of the nodal/paranodal cell adhesion molecule neurofascin were identified. All developed rapidly progressive tetraplegia. Cranial nerve deficits (100% vs 26%), autonomic dysfunction (75% vs 13%) and respiratory involvement (88% vs 14%) were more common than in seronegative patients. Four patients died despite treatment with one or more modalities of standard immunotherapy (intravenous immunoglobulin, steroids and/or plasmapheresis), whereas the four patients who later went on to receive the B cell-depleting therapy rituximab then began to show progressive functional improvements within weeks, became seronegative and ultimately became functionally independent.

**Conclusions:**

IgG_1_ pan-neurofascin antibodies define a very severe autoimmune neuropathy. We urgently recommend trials of targeted immunotherapy for this serologically classified patient group.

## Introduction

Guillain-Barré syndrome (GBS) is characterised by flaccid limb weakness, supressed deep tendon reflexes and a monophasic disease course reaching nadir within 4 weeks. Cranial nerve and autonomic dysfunction are common, and around 25% of affected individuals develop neuromuscular respiratory failure.^1^ Demyelinating and axonal subtypes are defined by neurophysiology.^2^ In chronic inflammatory demyelinating polyneuropathy (CIDP), disease activity and clinical progression continue for more than 8 weeks from onset.^3^

The node of Ranvier facilitates fast and efficient saltatory conduction along myelinated axons, which is reliant on the strict localisation of voltage-gated sodium channels and voltage-gated potassium channels at the node and juxtaparanode, respectively. This is ensured in part by cell adhesion molecules at the node (neurofascin-186 (NF186) and gliomedin) and paranode (contactin-1 (CNTN1), contactin-associated protein (Caspr1) and neurofascin-155 (NF155)).^4^

Pathology affecting the node, termed ‘nodo/paranodopathy’, has been linked to some forms of GBS,^5^ in which anti-ganglioside antibodies capable of inducing complement-mediated nodal injury are found.^6^ Recently, antibodies directed against nodal/paranodal proteins have been identified in patients meeting diagnostic criteria for CIDP.^4 7–12^

Herein, we describe eight patients with a very severe neuropathy associated with ‘pan-neurofascin’ (panNF) IgG_1_-subclass antibodies.

## Methods

From July 2017 to May 2020, we tested serum samples from 649 patients with suspected inflammatory neuropathies, and 210 controls, for IgG antibodies directed against nodal (NF186) and paranodal (NF155, CNTN1 and Caspr1) cell adhesion molecules, using a live, cell-based assay (CBA).^12^ A standardised request form was used to collect clinical data. Further methodological details are given in the online supplemental appendix. The data that support the findings of this study are available from the corresponding author, on reasonable request.

10.1136/jnnp-2021-326343.supp1 Supplementary data

## Results

Overall, 46 of 649 patients with suspected inflammatory neuropathies (7.1%) were positive for nodal/paranodal IgG-class antibodies. These antibodies were not detected in 210 controls (90 patients with other neurological diseases (20 with multiple sclerosis, 70 with antibody-positive central nervous system disorders) and 120 healthy individuals). Seropositive patients consisted of 17 (2.5%) with antibodies against NF155 alone, 1 (0.15%) with monospecific NF186 antibodies, 11 (1.6%) with CNTN1 antibodies alone, and 9 (1.3%) with CNTN1/Caspr1 complex antibodies. Patients with the latter two antibody specificities were included in previous studies.^11 13 14^

**Figure 1:**
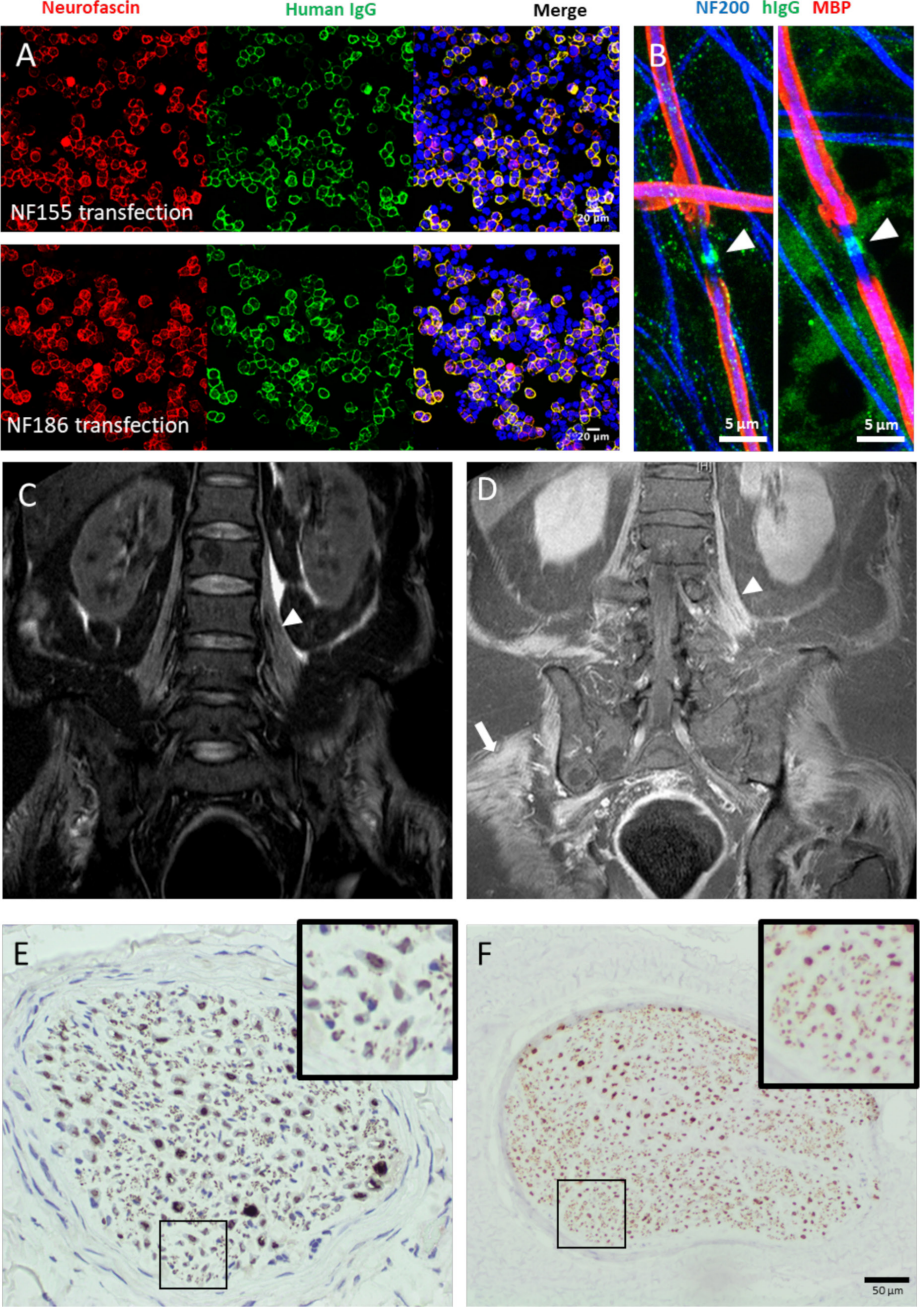
Serological, radiological and histological findings. (A) Cell-based assays using HEK293T cells transiently transfected to overexpress neurofascin-155 (NF155) (upper panels) or neurofascin-186 (NF186) (lower panels). Neurofascin (red) expression in the cell membrane is revealed by a commercial polyclonal antibody, and colocalises with human IgG (green) after exposure to acute-phase serum from the patients described in this series. (B) IgG (green) from two pan-neurofascin antibody-positive patient sera (left, P1; right, P6) is deposited at the node of Ranvier (arrowhead) after exposure to myelinating co-cultures. Axons (neurofilament-heavy, NF200, blue) were also observed weakly labelled with punctate IgG deposition in P1. Neither nodal or axonal labelling was observed in sera from healthy controls (data not shown). Myelin basic protein (MBP, red) defines the myelinated internode. (C, D) MRI of the lumbar spine, with coronal short tau inversion recovery (C) and post-contrast T1 (D) sequences, shows diffuse symmetric thickening and enhancement of lumbosacral plexus nerve roots (arrowhead), and enhancement of paraspinal, psoas, pelvic and proximal leg skeletal muscles (arrow). (E) Nerve biopsy from P6 stained for NFP shows reduced numbers of NFP positive axons (more clearly seen in the inset panels), and dense patches of staining, consistent with axonal degeneration. ‘Near normal’ NFP staining (F) is shown for comparison.

Eight patients (1.2%) had IgG antibodies which cross-reacted with both the nodal/axonal NF186 and NF140 isoforms, and paranodal/glial NF155 isoform (subsequently termed ‘panNF’) (figure 1A and online supplemental figure 1). These antibodies were exclusively IgG_1_, while IgG_3_ and/or IgG_4_-subclass antibodies with panNF reactivity were not detected. In contrast, using the same assays, IgG_1_ was exclusively detected in only two patients with NF155 monospecific antibodies, and in none of the CNTN1 or CNTN1/Caspr1-positive patients. IgG_4_ was the dominant subclass in the majority of patients in all the other antibody groups, though all other subclass antibodies could occasionally be detected at lower intensities (online supplemental table 1 and figure 1). All patients showed only one of the five distinct patterns of serological reactivity. Specifically, all eight panNF-positive sera and all 17 NF155-positive sera were negative for CNTN1 and CNTN1/Caspr1 antibodies, all CNTN1 and CNTN1/Caspr1-positive patients were negative for both NF155 and NF186 antibodies, and all CNTN1/Caspr1-positive patients were also negative for CNTN1 antibodies.

As the clinical associations of IgG_1_-subclass panNF antibodies have not been reported, we sought to assess the characteristics of this serologically defined cohort and compare these with seronegative patients and those seropositive for other nodal/paranodal antibodies, particularly focusing on those with NF155 monospecific antibodies only.

### Clinical features

Clinical details were available for all eight panNF antibody-positive patients, 15 of 17 NF155, 11 of 11 CNTN1, 8 of 9 CNTN1/Caspr1-positive patients, and 194 of 603 seronegative cases (table 1). The median age of this patient cohort was 68.5 years (range 43–78), and the majority male (75%). Overall, there were significant differences between these groups in the frequency of patients initially diagnosed with GBS (p=0.03), experiencing tremor (p=0.03) or neuropathic pain (p=0.01), or developing cranial nerve palsies, autonomic dysfunction, respiratory involvement or episodes of acute deterioration (all p<0.001, multiple Χ^2^ tests). There were no significant differences in the frequencies of patients with ataxia (p=0.26) or MRI plexus/nerve root abnormalities (p=0.09, Χ^2^ tests).

**Table 1:** Clinical features of patients with pan-neurofascin (panNF) antibodies and comparison with patients with neurofascin-155 (NF155), CNTN1, CNTN1/Caspr1 antibodies, and seronegative cohorts

Clinical feature (n, %)	PanNF (n=8)	NF155 (n=15)	CNTN1 (n=11)	CNTN1/Caspr1 (n=8)	Seronegative (n=194)	OR vs NF155	95% CI	OR vs seronegative	95% CI
Initial clinical diagnosis of GBS	5/8 (63%)	3/15 (20%)	3/11 (27%)	4/7 (57%)	38/185 (21%)	6.7	1.1 to 34.5	6.5	1.6 to 24.9
Acute/subacute progression	8/8 (100%)	7/15 (47%)	4/11 (36%)	5/7 (71%)	56/184 (30%)	∞	2.0 to ∞	∞	4.8 to ∞
Ataxia	3/8 (38%)	7/15 (47%)	7/11 (64%)	5/7 (71%)	62/158 (39%)	0.7	0.1 to 3.4	0.9	0.2 to 3.7
Tremor	0/8 (0%)	5/15 (33%)	3/11 (27%)	3/7 (43%)	39/154 (25%)	0	0 to 1.3	0	0 to 1.4
Neuropathic pain	4/8 (50%)	1/15 (7%)	7/11 (64%)	5/7 (71%)	49/134 (37%)	14	1.3 to 180	1.7	0.5 to 6.2
Cranial nerve palsy	8/8 (100%)	5/15 (33%)	5/11 (45%)	1/7 (14%)	41/156 (26%)	∞	3.3 to ∞	∞	5.7 to ∞
Autonomic dysfunction	6/8 (75%)	0/15 (0%)	2/11 (18%)	0/2	9/71 (13%)	∞	6.1 to ∞	20.7	3.9 to 105.4
Respiratory involvement	7/8 (88%)	0/15 (0%)	3/11 (27%)	0/7	25/185 (14%)	∞	9.9 to ∞	44.8	7.3 to 506.3
Nephrotic syndrome	3/8 (38%)	0/15 (0%)	9/11 (82%)	0/7	5/147 (3%)	∞	1.9 to ∞	17	3.5 to 73.7
MRI plexus/root abnormalities	2/4 (50%)	2/7 (29%)	2/7 (29%)	5/6 (83%)	15/53 (28%)	6.7	1.1 to 34.5	6.5	1.6 to 24.9
Nadir mRS >4	8/8 (100%)	3/15 (20%)	4/11 (36%)	2/7 (29%)	38/185 (21%)				
						**Significant vs NF155**	**P value**	**Significant vs seronegative**	**P value**
Nadir mRS (median, range)	5.5 (5–6)	4 (2–5)	4 (2–6)	4 (4–5)	3 (1–5)	**	0.006	***	<0.001
CSF protein (g/L) (median, range)	0.48 (0.34–0.62)	1.65 (0.61–7.05)	2 (0.24–5.9)	2.7 (0.91–4.46)	0.87 (0.18–6.0)	***	<0.001	*	0.04

The 95% CI of the OR was calculated by the Baptista-Pike method. Nadir mRS and CSF protein were compared by a two-tailed Kruskal-Wallis test with Dunn’s correction for multiple comparisons. The patients with CNTN1 and CNTN1/Caspr1 antibodies were included in previous studies.^11 13^

*p<0.05, **p<0.01, *** p<0.001.

Caspr1, contactin-associated protein; CNTN1, contactin-1; CSF, cerebrospinal fluid; GBS, Guillain-Barré syndrome; mRS, modified Rankin Scale.

PanNF antibody-positive patients were all very severely affected and had rapidly developed profound tetraplegia. Compared with seronegative patients, they were more likely to have presented following acute or subacute deterioration (OR ∞, 95% CI 4.8 to ∞), and to have received an initial clinical diagnosis of GBS (OR 6.5, 95% CI 1.6 to 24.9). Nadir modified Rankin Scale (mRS) scores (median 5.5, range 5–6) were significantly higher than those of the NF155 monospecific antibody-positive (median 4, range 2–5, p=0.005), CNTN1 antibody-positive (median 4, range 2–6, p=0.05) and seronegative patients (median 3, range 1–5, p<0.001), but non-significantly higher than those with CNTN1/Caspr1 complex antibodies (median 4, range 4–5, p=0.59, Kruskal-Wallis test with Dunn’s correction for multiple comparisons) (table 1 and online supplemental figure 2A). Cranial nerve palsies (100%), autonomic dysfunction (75%) and respiratory compromise (88%) were also more frequent than in seronegative patients and those with other nodal/paranodal antibodies (table 1 and online supplemental table 2). A small number of patients (2 of 7) had evidence of papilloedema. Concurrent presentation with nephrotic syndrome was notable (38%) but not as frequent as reported in the CNTN1 antibody-positive group (82%).^11^ Ataxia (3 of 8) and neuropathic pain (4 of 8) were occasional features in panNF-positive patients, although less common than in CNTN1 and CNTN1/Caspr1 antibody-positive patients. Clinical vignettes for patients 1, 5 and 6 are given in the online supplemental appendix. Patient 4 was described in a recent case report.^15^

### Laboratory findings

During work-up of their neuropathy or shortly thereafter, two panNF antibody-positive patients were found to have an IgG-lambda paraprotein and were subsequently diagnosed with lymphoproliferative disorders (Hodgkin’s lymphoma and chronic lymphocytic leukaemia). A third (P8—online supplemental table 2) was found to have a clonal urinary lambda light chain, without a serum paraprotein, that was not further investigated prior to his death. Three patients had features of nephrotic syndrome (peripheral oedema and hypoalbuminaemia) which had developed in parallel with their neuropathy, and, in two, urinary protein levels were analysed and nephrotic range proteinuria confirmed. All patients were otherwise negative for standard neuropathy screening bloods, including anti-GM1 and GQ1b-ganglioside antibodies. In panNF-positive patients, cerebrospinal fluid (CSF) protein was either normal or only marginally elevated at presentation (evaluated in all seven patients tested within 14 days of onset, median 0.51 g/L, range 0.34–0.62), non-significantly lower than that of the seronegative patients (median 0.87 g/L, range 0.18–6, p=0.09) and significantly lower than in all other seropositive cohorts (p=0.007 vs CNTN1 and p<0.001 vs NF155 or CNTN1/Caspr1, Kruskal-Wallis with Dunn’s correction for multiple comparisons) (online supplemental figure 2D). CSF white cell counts were invariably normal (range 1–3/μL) with unremarkable cytology and flow cytometry.

Using the CBA, all patients had IgG_1_-subclass antibodies reactive against both paranodal NF155 and nodal NF186 and NF140 isoforms, and were negative on IgG_2_, IgG_3_ and IgG_4_-subclass-specific assays (online supplemental table 1 and online supplemental figure 1B). Endpoint titres ranged from 1:400 to 1:3200 (online supplemental figure 4A). Six of seven patients tested were also positive on a neurofascin ELISA, although the endpoint titres were consistently lower than those obtained by CBA. In contrast, ELISA appeared to be slightly more sensitive for the detection of CNTN1 antibodies (online supplemental figure 3C). Detection of subclass-specific antibodies by ELISA was also less sensitive than with CBA, with an IgG_1_ signal above background only being detected in only two patient samples (online supplemental figure 4B).

Five of the seven panNF sera tested showed nodal binding, and two additional axonal binding, in live, myelinating co-cultures (figure 1B and online supplemental figure 4A) generated from human-induced pluripotent stem cell-derived neurons. These sera showed a pattern distinct from that seen with NF155 monospecific sera (online supplemental figure 4A).^16^ No patients had IgG_4_ panNF antibodies or developed these during follow-up.

The antigen specificity of panNF antibodies detected in patient sera was confirmed by pre-adsorption assays in CBA and myelinating co-cultures. Pre-incubation of sera with soluble NF155 or NF186 protein abrogated cell membrane IgG labelling in trasiently transfected human embryonic kidney (HEK293) cells (online supplemental figure 1C, D), and nodal/axonal labelling in co-cultures (online supplemental figure 4).

ELISA was performed to assess whether panNF antibodies in the sera of the two patients with IgG-lambda paraproteins exclusively used lambda light chains. In each case, both kappa and lambda light chain-containing panNF antibodies were detected (data not shown).

### Neurophysiology

Neurophysiological results were available for six of eight patients. In one, the nerves were inexcitable when first assessed 2 weeks after onset. In four of six, conduction slowing on initial studies was considered to indicate demyelination. However, five of six showed conduction block without temporal dispersion, suggestive of nodal pathology.^5^ In three, follow-up studies 3–4 weeks later revealed very reduced or unrecordable compound muscle action potentials and electromyographic findings consistent with severe axonal degeneration. Detailed neurophysiological results are given in online supplemental table 3.

### Imaging

Four patients were examined by MRI. In one, symmetric enhancement and thickening of the lumbosacral plexus nerve roots, as well as enhancement of paraspinal, pelvic and proximal lower limb muscles, were observed. T2 hyperintensities of the brachial plexus and L5–S2 roots but no thickening or enhancement were seen in another (figure 1C, D).

### Histology

Nerve biopsy was performed in two patients. In both cases, this demonstrated axonal loss, without any features of cellular infiltration, inflammation, segmental demyelination, amyloid or vasculitis (figure 1E and online supplemental figure 5). Electron microscopy was performed in one patient and did not show any evidence of paranodal retraction/detachment. One patient had a necrotising myopathy on muscle biopsy with a normal creatine kinase.

### Treatment and outcome

All patients received intravenous immunoglobulin (IVIg) 2 g/kg over 5 days. In six cases, this was not associated with any perceptible benefit. In two cases, there was a minor and/or transient neurological improvement. Six patients received at least one cycle of plasma exchange (PLEx), with three patients showing slight but non-sustained neurological recovery. Further detail and physician reported assessments of response are given in online supplemental tables 2 and 4, as well as treatment time lines in online supplemental figure 6. Four patients died. One suffered a cardiorespiratory arrest 8 days after presentation, and in the absence of recovery of cortical function, ventilatory support was withdrawn 10 days later. In another, recurrent pulmonary infections and the absence of any neurological recovery after IVIg and two cycles of PLEx led to the withdrawal of ventilatory support on day 108. A third patient, with comorbid metastatic breast cancer, declined artificial ventilation, having failed to respond to steroids, IVIg, PLEx and cyclophosphamide, and died on day 93. Most recently, during the COVID-19 pandemic, intensive care and mechanical ventilation were not deemed appropriate for one patient who was SARS-CoV-2 PCR negative, but developed increasing breathlessness and tachypnoea on day 12, and died 48 hours later. In both patients with nephrotic range proteinuria, this was still apparent when last measured shortly before their death.

After initial rounds of treatment with IVIg, PLEx and steroids, following which panNF antibodies were detected, the remaining four patients received rituximab 3–4 months (1 g repeated after 2 weeks) into their illness, following no or minimal and transient apparent responses to other therapies, as assessed by their treating physicians. All four patients were found to be panNF antibody positive from their initial rounds of therapy, prior to rituximab treatment being started. Two patients additionally received combination chemotherapy for newly diagnosed haematological disorders. In one (P4), this began concurrently with rituximab, and was started (P3) 5 months later in the other. All four rituximab-treated patients made progressive functional improvements, regained independent mobility and were ultimately discharged home, often via a rehabilitation facility. All showed improvements of at least 3 points on the mRS within 6 months of rituximab treatment, and two became asymptomatic by 9 months (online supplemental figure 2B). In the one rituximab-treated patient with nephrotic syndrome, the serum albumin normalised in parallel with neurological improvement. In all four patients, neurofascin antibodies were negative when retested 4–11 months after rituximab. One of these four patients, having returned almost to his baseline (mRS=1, minimal residual symptoms) had a return of motor and sensory symptoms, approximately 18 months after receiving a single cycle of rituximab. He developed marked arm weakness and became immobile over a few weeks, and redeveloped hypoalbuminaemia in parallel. At this stage, panNF antibodies were again positive, though at low titre (1:100 and 1:200). Again, IgG_1_ was the dominant subclass. He was retreated with steroids and a second cycle of rituximab, and again improved, regaining independence.

## Discussion

This is the first reported series of patients with IgG_1_-subclass, panNF antibodies. These antibodies are shown to be associated with an extremely severe and rapidly progressive neuropathy.

To date, antibodies directed against nodal and paranodal cell adhesion molecules have largely been described in patients meeting diagnostic for CIDP. In this context, IgG_4_ antibodies have been reported as pathologically important markers of a clinically distinct CIDP subgroup, whereas patients with only non-IgG_4_-subclass antibodies were found to be indistinguishable from seronegative individuals.^17^ In our series, patients with IgG_1_-subclass panNF antibodies, who had significantly different clinical features and disease severity compared with identically identified seronegative controls, were more likely to have received an initial clinical diagnosis of GBS.

In the five panNF patients in our series diagnosed with GBS, there was no clinical basis to reclassify their neuropathy as CIDP. None of these patients had clinical evidence of progressive neuropathy beyond 4 weeks from onset. Three showed one or two transient fluctuations after treatment, though all within 8 weeks of onset.^18^ The three other patients met the clinical and electrodiagnostic criteria for definite CIDP,^3^ yet their neuropathy was likely to represent a nodo/paranodopathy rather than being primarily demyelinating.

At present, the distinction between GBS and CIDP can only be confidently drawn when ongoing deterioration is seen more than 8 weeks after onset.^18^ This criterion is of little to no use in informing therapeutic decisions during the early phases of a rapidly progressive, but potentially chronically persistent, and treatable autoimmune/inflammatory neuropathy. As the immunopathological process in GBS is typically conceptualised as monophasic and short lived, a diagnosis of GBS provides little impetus to give immunomodulatory therapy outside of the acute phase. We therefore believe that testing for nodal/paranodal antibodies is justified in any patient with a GBS-like presentation, and in particular in severe cases poorly responsive to standard immunotherapy. Of note, 36.6% of our nodal/paranodal antibody-positive cohort overall received an initial clinical diagnosis of GBS.

The improvements after rituximab seen here occurred several months into the illness, after responses to steroids, IVIg and/or PLEx were deemed inadequate. Our observations suggest that a more persistent autoimmune response can potentially drive ongoing axonal loss, and prevent recovery, in patients whose initial presentation nonetheless resembles GBS, and in whom clinical deterioration greater than 8 weeks from onset is not always apparent, preventing a diagnosis of CIDP. They also raise the possibility that treatment approaches that lead to more prolonged suppressions of antibody titres may be required for sustained neurological improvement in patients with nodal/paranodal antibodies.^12^ Serological results and biomarkers of ongoing peripheral nerve injury may in future prove to have greater utility in guiding these treatment decisions and may ultimately supersede clinical categorisation. The earlier use of potent immunotherapies with longer duration of action in such patients may be more effective in reducing long-term disability.

In this observational study, patients received multiple different therapies at different time points. We cannot, therefore, provide any clear evidence on the efficacy of any particular therapy in this setting. It is possible that the four patients who survived long enough to be treated with rituximab would have started to improve even if this therapy had not been given. We also cannot determine to what extent chemotherapy for the underlying haematological malignancies contributed to neurological recovery in two of the patients. Future trials should address whether the earlier use of targeted immunotherapy in such serologically defined cohorts could ameliorate the very severe disease course seen here.

Cancer is rarely associated with GBS/CIDP,^19^ though onconeural antibodies have not previously been identified. The frequency of IgG/lambda paraprotein-associated lymphoproliferative disorders and solid organ malignancy (4 of 8 overall) in this cohort suggests that panNF antibodies may be responsible for some such cases.

Antibodies against the paranodal isoform NF155 have been linked to ‘atypical CIDP’. NF155 monospecific seropositive individuals are often younger men with predominantly distal weakness, sensory ataxia and tremor. NF155 autoantibodies are predominantly of the non-complement-fixing IgG_4_ subclass and the response to IVIg is typically poor.^8^ Antibodies against nodal isoforms of NF140/186 have been described in 12 patients so far. Four out of the five originally described patients were diagnosed with CIDP^10^ and had predominantly IgG_4_-subclass antibodies, but three of four improved after IVIg. The remaining patient had IgG_3_-subclass antibodies and did not improve after IVIg, but did so after steroids and PLEx. Similar to our cohort, two of five were found to have nephrotic syndrome. This complication appears less common than in patients with CNTN1 antibodies,^11^ and may be explained by the expression of NF186 by glomerular podocytes as well as neurons.^20^ A further five severely affected neurofascin IgG_3_ seropositive patients with a reported poor response to standard therapies have more recently been described. In all but one case, antibodies cross-reacted with both NF140/186 and NF155 in CBAs, as in our cohort. One patient treated with rituximab subsequently improved, as did another, apparently spontaneously, starting 3 months into his illness.^21–23^ The patients with IgG_3_ NF140/186 and panNF antibodies previously reported seem most similar to our cohort. The differences in the panNF antibody–subclass distribution detected in our study may be due to technical factors related to the diagnostic assays. The secondary antibodies used here were shown to recognise recombinant human IgG of the relevant subclass in ELISA, did not cross-react with any of the other subclasses and were all detected with the same tertiary antibody. Whereas most other studies used ELISA, we found CBAs to be more sensitive in the detection of panNF antibodies and to determine subclass (online supplemental figure 3). This has also been reported with other antigenic targets^24^ and should stimulate calls for a multicentre, interlaboratory, blinded comparison study of solid-state and live cell-based nodal/paranodal antibody assays.

We have shown that the antibodies from these patients’ sera specifically target three neurofascin isoforms, and that pre-adsorption of panNF sera with neurofascin isoforms 155 or 186 abrogates IgG binding to cells expressing NF186 or NF155, and nodes of Ranvier within a live neuronal culture. This suggests the panNF antibodies recognise an epitope common to both isoforms. NF155, NF186 and NF140 are isoforms of the same protein that differ in the arrangement and composition of their fibronectin domains. Previous studies have shown that panNF antibodies specifically recognise the immunoglobulin domain shared between isoforms,^10 23^ in contrast to NF155 antibodies which require the third fibronectin domain unique to this isoform in order to bind.^25 26^

Whether panNF antibodies are pathogenic or simply an epiphenomenon remains to be determined. The predominance of the IgG_1_ subclass in this panNF patient cohort suggests complement may play a potential role in antibody pathogenicity. This important question should be addressed in future studies.

In summary, the observations herein provide further rationale for nodal/paranodal antibody testing in GBS-like presentations. They highlight the importance of testing against *both* glial and neuronal neurofascin isoforms (to distinguish panNF from NF155 monospecific antibodies) and determining the IgG subclass, as this may also influence the clinical phenotype and response to treatment. We advocate that such testing should increasingly form part of the routine diagnostic process. If it is not feasible to test nodal/paranodal antibodies in all GBS-like presentations, we believe antibody testing could be prioritised for those with severe disease, especially if there are no signs of response to the first round of treatment, and certainly if there is evidence of nephrotic syndrome or any suggestion of a more chronic autoimmune neuropathic process.

The possible benefit of rituximab in patients with panNF antibodies reported here should be evaluated in a well-conducted clinical trial, the design of which must consider the potentially grave outcome in this serologically defined cohort.

## Data Availability

Data are available upon reasonable request.
