# Supplementary material for: IgG1 pan-neurofascin antibodies identify a severe yet treatable neuropathy with a high mortality
Source: J Neurol Neurosurg Psychiatry. 2021 Aug 16;92(10):1089–95. doi: 10.1136/jnnp-2021-326343 (PMC8458075; doi:10.1136/jnnp-2021-326343)
Supplement: Supplementary data [file jnnp-2021-326343supp001.pdf]

Supplementary Material

Table of Contents

Supplementary methods 2

Request Form for Paranodal / Nodal Antibody Testing 4

Supplementary Table 1 - Subclass distribution of pan-neurofascin versus neurofascin-155, contactin-1 and contactin1/caspr1 complex reactive antibodies 6

Supplementary Table 2 - Clinical features of pan-neurofascin antibody positive patients 7

Supplementary Table 3 - Detailed neurophysiological findings 8

Supplementary Table 4 - Physician reported treatment response by serological group 9

Supplementary Figure 1 - Comparison between neurofascin-155 monospecific and pan-neurofascin serum reactivity on cell-based assays 10

Supplementary Figure 2 - Modified Rankin scores and CSF protein levels 11

Supplementary Figure 3 - Subclass specificity and comparison between CBA and ELISA 13

Supplementary Figure 4 – Pan-neurofascin serum is specific to NF155 and NF186 in live myelinating co-cultures 14

Supplementary Figure 5 - Teased nerve fibres 15

Supplementary Figure 6 – Treatment timelines 16

Case Vignettes 17

Online-only References 19

Fehmi J, *et al. J Neurol Neurosurg Psychiatry* 2021; 92:1089–1095. doi: 10.1136/jnnp-2021-326343

Supplementary methods

Nodal/paranodal antibody cell-based assays

All sera were initially screened using a live, transiently transfected cell-based assay (CBA), following previously described methods with slight modification.[1] Human embryonic kidney 293T ("HEK") cells were plated at a density of 75,000 cells/13mm coverslip, and mono-transfected with either neurofascin-155 (NF155, *NFASC*) (RC228652, Origene), neurofascin-186 (NF186, courtesy of Jerome Devaux, University of Marseille), or co-transfected with contactin-1 (*CNTN1*, EXA1153-MO29 Genecopoeia, Maryland, US) and contactin-associated protein ('1') (*CASPR1*, EXMO417-MO2 Genecopoeia, Maryland, US), diluted in Jet-PEI transfection reagent (101-10; Polyplus). Patient sera were screened using a dilution to 1:100 in DMEM/1% bovine serum albumin (BSA) for incubation with live neurofascin transfected cells (1h at 37□C), then titred out to 1:6400. Co-incubation with commercial chicken anti-neurofascin primary antibody, (1:1000) (Cat no. AF3235; R&D Systems, Bio-Techne) was used to confirm successful transfection and to assess for co-localisation with any bound human IgG. Cells were then fixed with 4% paraformaldehyde and secondary antibody incubation was with goat anti-human IgG-Fc specific-Alexa Fluor 488 (1:750) (Cat no. H10120; Life Tech) and goat anti-Chicken Alexa Fluor 546 1:1000 (Cat no. A11040; Life Tech). To determine antibody subclass unconjugated mouse anti-human IgG subclass 1-4 antibodies were used at 1:100 (Cat nos. I2513, I25635, I7260 I7385; Sigma-Aldrich, Merck) with the fluorescently tagged tertiary antibody goat anti-mouse Alexa Fluor 488 (1:750) (Cat no. A11029; Life Tech).

ELISA

ELISA was performed as previously described.[2] In brief, Nunc Maxisorp ELISA plates (Fisher Scientific) were coated with 100 ml/well of PBS containing human recombinant neurofascin-155 (NF155) (8208-NF, R&D systems), NF186 (TP329070, OriGene Technologies) or CNTN1 (10383-H08H, Sino Biological Inc) at 1 mg/ml. Blank wells were incubated with carrier only. After overnight incubation at 4°C, the coating solution was tipped off and wells were blocked with 5% milk for 1h at room temperature. Sera were incubated for 1h at room temperature, and initially screened at 1:100 dilution in 5% milk. Following a wash step through 5 changes of PBS, anti-human IgG (Fc-specific) peroxidase-conjugated anti-human IgG (A0170, Sigma) was applied at 1:3000 in 5% milk and incubated for 1h at room temperature, then washed as before. The detection reaction was performed using o-Phenylenediamine dihydrochloride (OPD, Sigma), applied for 20 minutes in the dark, then terminated with 4M H_2SO_4. Optical densities were measured at 492nm. Wells with ODs greater than 0.1 above uncoated control wells were considered positive. The end-point titre of positive samples was then assessed by serial doubling dilution from 1:100 to 1:6400. The end-point titre was defined as the highest dilution with an OD greater than 0.1 above the uncoated control. To assess the specificity of the subclass specific secondary antibodies used in the CBA, recombinant human IgG subclass proteins (HCA049, HCA50, HCA178, HCA193, HCA194, HCA195, Bio-Rad) were coated as above, and then exposed to the unconjugated mouse anti-human IgG subclass 1-4 antibodies for 1h at room temperature (IgG1 1:1000, IgG2 1:250, IgG3 1:100, IgG4 1:250). Following a wash step, a HRP anti-mouse-IgG antibody (A4416, Sigma) was applied at 1:1000 for 1h, washed off, and binding detected as before. A standard curve was created for each subclass antibody using duplicate wells coated with the recombinant subclass proteins on the same plate as patient sera, and the limit of the blank (highest detectable analyte in duplicate blank wells) and limit of detection (lowest analyte concentration distinguishable from the blank) for each subclass were calculated.[3] Optical densities 5 standard deviations of a low concentration analyte above the limit of the blank were considered positive.

Collection of clinical data

A request form (over page) was sent to all clinicians submitting samples for nodal/paranodal antibody testing and initial information was requested before test results were known. Clinical data was obtained from all 8 patients with pan-neurofascin antibodies, 15/17 with neurofascin-155 antibodies, and 194/606 seronegative individuals. Two attempts were made to collect missing data by re-contacting referring clinicians directly. All patients without clinical data returns following these efforts were omitted from the subsequent analysis. Follow up times varied from 3 years to 2 months for the antibody positive cohort (average 19 months), with the shortest follow up for the surviving pan-neurofascin patient being 9 months. Nodal/paranodal and seronegative neuropathy patients seen in Oxford were recruited to an observational study. This study was approved by the NHS National Research Ethics Service Committee (South Central – Oxford A, 14/SC/0280). The use of de-identified clinical data from other patients to audit the relative value of available and novel methods to

determine autoantibodies, based on the gold standard of immune-mediated clinical phenotypes, was approved by the Oxford University Hospitals NHS Trust clinical audit leads (ID 5106).

Analysis of clinical associations

Contingency data for the presence or absence of 10 core clinical features (diagnostic category, onset/progression, ataxia, tremor, neuropathic pain, cranial nerve palsy, autonomic dysfunction, respiratory involvement, nephrotic syndrome, and MRI abnormalities) were analysed by Chi-square tests without family-wise correction for error rate. For subsequent comparisons between panNF positive patients, NF155 antibody positive patients, and seronegative patients, Fisher's exact test was used. Point estimates of the odds ratio are reported, with 95% confidence intervals calculated by the Baptista-Pike method. The widths of the intervals have not been adjusted for multiplicity and as such the inferences drawn may not be reproducible. The nadir modified Rankin score and CSF protein level at diagnosis were compared by the Kruskal-Wallis test with Dunn's correction for multiple comparisons.

Preparation and immunofluorescence of myelinating co-cultures

Sera were assessed for topographical binding using myelinated co-cultures. These were generated using sensory neurons derived from induced pluripotent stem cells (iPSC) according to a previously described protocol.[4] iPSCs were differentiated into neurons over 10 days, and myelinated with rat Schwann cells after four weeks to establish mature myelinated co-cultures. For immunolabelling, these were incubated with the patient's sera, diluted at 1:100, for 1h at 37□C, washed in 3 changes of DMEM/HEPES, then fixed with 2% PFA before incubation with secondary antibody goat anti-human IgG AF488 (Cat no. A11013: ThermoFisher Scientific). The cultures were then permeabilised with ice-cold methanol before co-staining was used to localise specific peripheral nerve subdomains. This involved either chicken anti-Neurofilament 200 (1:10,000) (Cat no. 4680; Abcam) if axons were being labelled, or rabbit anti-Caspr (1:1000, gift from Dr Manzoor Bhat, UT Health Science Center San Antonio) if paranodes were labelled, and rat anti-myelin basic protein (1:500) (Cat no. 7349; Abcam). The secondary antibodies combinations then used were biotinylated goat anti-chicken IgY (1:500) (Cat no. BA9010; Vector lab) and goat anti-rat IgG Alexa Fluor 546 (1:1000) (Cat no. A11081 Life Tech), followed by Streptavidin pacific blue (1:500) (Cat no. S11222 Life Tech), or goat anti-rabbit IgG Alexa Fluor 546 (1:400) (Cat no. A11010: ThermoFisher Scientific) and biotinylated goat anti-rat IgG (1:1000) (Cat no. BA9400; Vector lab), followed by Streptavidin pacific blue. Fluorescence images of IgG nodal labelling in myelinating cultures were acquired on a laser scanning confocal microscope (LSM 700, Zeiss) using the x63 or x20 objectives. 10-15 z-sections at 0.5 µm interval were exported as maximum projection images. Brightness and contrast were adjusted for presentation.

Pre-adsorption

Pre-adsorption assays were performed by first incubating sera with 1ug of the same recombinant NF155 or NF186 protein used in the ELISA, at 4°C overnight on a rotating mixer. Pre-incubated sera was then used in the myelinating co-culture and cell-based assays in the same way as previously described for non-pre-adsorbed sera.

Fehmi J*, et al. J Neurol Neurosurg Psychiatry* 2021; 92:1089–1095. doi: 10.1136/jnnp-2021-326343

Request Form for Paranodal / Nodal Antibody Testing

Clinical Data

Date of neuropathy onset: Age at diagnosis:

☐ Prodromal illness/trigger (please specify):
Start date for prodrome/trigger:

Initial diagnosis
☐ GBS ☐ Typical CIDP ☐ MMN
☐ Atypical CIDP / Other (specify):

Current diagnosis
☐ GBS ☐ CIDP ☐ MMN
☐ Atypical CIDP / Other (specify):

If the current diagnosis is GBS, CIDP, or MMN please answer the following (tick all that apply):

Clinical course
☐ Relapsing-remitting ☐ Progressive ☐ Monophasic

Onset / progression
☐ Acute (<4 weeks) ☐ Subacute (4-8 weeks) ☐ Chronic (>8 weeks)

Weakness (Yes/No) (Tick all that apply)
☐ Arms
☐ Proximal ☐ Distal
☐ Asymmetric ☐ Symmetric

☐ Legs
☐ Proximal ☐ Distal
☐ Asymmetric ☐ Symmetric

Sensory deficit (Tick all that apply)
☐ Arms
☐ Vibration ☐ Pinprick ☐ JPS

☐ Legs
☐ Vibration ☐ Pinprick ☐ JPS

☐ Ataxia ☐ Tremor ☐ Neuropathic pain 0 /10 Severity (1-10)

Reflexes
☐ Absent ☐ Decreased ☐ Normal ☐ Brisk

☐ Cranial nerve involvement (specify)

☐ Autonomic involvement (please specify)

Respiratory involvement ☐ Current ☐ Previous ☐ None

Evidence of nephrotic syndrome
☐ Proteinuria (level:) ☐ Oedema ☐ Not assessed
☐ Hypoalbuminaemia (nadir level: g/L) ☐ None

Severity
Modified Rankin score (at nadir): 1 ☐ 2 ☐ 3 ☐ 4 ☐ 5 ☐ 6 ☐

Fehmi J, *et al. J Neurol Neurosurg Psychiatry* 2021; 92:1089–1095. doi: 10.1136/jnnp-2021-326343

Investigations

☐ CSF (at diagnosis) Date:
Protein: g/L WCC: RCC:
OCBs: (select from drop down menu) Other:

Neurophysiology
Overall impression: ☐ Demyelinating ☐ Axonal ☐ Mixed Other (specify)

☐ Motor involvement (describe core features):
☐ Sensory involvement (describe core features):

Other Antibodies
Gangliosides ☐ Positive ☐ Negative ☐ Not done
Anti-MAG ☐ Positive ☐ Negative ☐ Not done

Paraprotein ☐ Positive ☐ Negative ☐ Not done
☐ IgG ☐ IgM ☐ IgA ☐ Kappa ☐ Lambda
Level: g/L

Imaging
MRI lumbar roots ☐ Abnormal ☐ Normal ☐ Not done
Specify:

Treatment and Outcome

	Trialled		Response: Good	Partial	None	Worse
**IVIg**	Yes ☐	No ☐	☐	☐	☐	☐
**Steroids**	Yes ☐	No ☐	☐	☐	☐	☐
**Plasma Ex.**	Yes ☐	No ☐	☐	☐	☐	☐
**Other** (specify)			☐	☐	☐	☐
**Other** (specify)			☐	☐	☐	☐

Current Disease Activity

1.Cure: ≥5 years off treatment
☐ A. Normal examination
☐ B. Abnormal examination, stable/improving

2. Remission: <5 years off treatment
☐ A. Normal examination
☐ B. Abnormal examination, stable/improving

3. Stable active disease: ≥1 year, on treatment
☐ A. Normal examination
☐ B. Abnormal examination, stable/improving

4. Improvement: ≥3 months <1 year, on Treatment
☐ A. Normal examination
☐ B. Abnormal examination, stable/improving

5. Unstable active disease: abnormal examination with progressive or relapsing course*
☐ A. Treatment naïve or <3 months
☐ B. Off treatment
☐ C. On treatment

Modified Rankin score (at best post treatment):
0 ☐ 1 ☐ 2 ☐ 3 ☐ 4 ☐ 5 ☐ 6 ☐

Fehmi J, *et al. J Neurol Neurosurg Psychiatry* 2021; 92:1089–1095. doi: 10.1136/jnnp-2021-326343

	Dominant subclass				Subclasses detected			
	PanNF (n=8)	NF155 (n=17)	CNTN1 (n=11)	CNTN1/Caspr1 (n=9)	PanNF (n=8)	NF155 (n=15)	CNTN1 (n=11)	CNTN1/Caspr1 (n=9)
None	0	1	0	0	-	-	-	-
IgG1	8	4	1	2	8	11	8	7
IgG2	0	0	0	0	0	10	6	4
IgG3	0	0	0	0	0	1	4	1
IgG4	0	9	6	5	0	14	11	8
IgG4=1	0	2	4	1	-	-	-	-
IgG4=2	0	1	0	1	-	-	-	-

Supplementary Table 1 - Subclass distribution of pan-neurofascin versus neurofascin-155, contactin-1 and contactin1/caspr1 complex reactive antibodies

In all cases, IgG1 was the only subclass detected for pan-neurofascin antibodies. In contrast, with other antibody groups, IgG4 was most often the dominant subclass detected, but IgG1 and to a lesser extent IgG2 were also frequently observed.

Age at onset / Gender	Clinical									Investigations				Treatment (response)					Outcome
	**Initial Clinical Diagnosis**	**Motor/ Sensory**	**CN palsy**	**Autonomic**	**Ataxia**	**Pain**	**Associated conditions**	**ITU / MV**	**Nadir mRS**	**Para-protein**	**NCS (Overall impression)**	**Nerve biopsy**	**Anti-NF155/ 186 Titre (CBA)**	**Steroids**	**IVIg**	**PLEx**	**RTX**	**Other**	
P1	GBS	M&S	✓[a]	✓	×	✓	Nephrotic syndrome	✓	6	ND	Inexcitable	×	1:6400/ 1:3200	×	✓ (red)	✓ (red)	×	×	Death (Day 110)
P2	CIDP	Motor only	✓[b]	✓	×	✓	Breast Cancer	✓ (declined MV)	6	ND	Demyelinating	×	1:3200/ 1:3200	✓ (red)	✓ (yellow)	✓ (red)	×	CYC	Death (Day 93)
P3	CIDP	M&S	✓[c]	×	×	×	Hodgkin lymphoma	✓	5	IgG-Lambda	Demyelinating	Axonal loss	1:3200/ 1:1600	✓ (red)	✓ (red)	✓ (yellow)	✓ (green)	ChIVPP	mRS 2 (1 year post RTX)
P4	Atypical MMN	Motor only	✓[d]	×	×	×	CLL	×	5	IgG-Lambda	Axonal	×	1:800/ 1:1800	✓ (red)	✓ (red)	✓ (yellow)	✓ (green)	CYC FDB	mRS 0 (9 months post RTX)
P5	GBS	M&S	✓[e]	✓	✓	×	No	✓	5	ND	Demyelinating	×	1:400/ 1:800	✓ (yellow)	✓ (yellow)	✓ (yellow)	✓ (green)	×	mRS 0 (9 months post RTX)
P6	GBS	M&S	✓[f]	✓	✓	✓	Nephrotic syndrome	✓	5	ND	Mixed	Axonal loss	1:3200/ 1:6400	✓ (yellow)	✓ (red)	✓ (red)	✓ (green)	×	mRS 1 (18 months post RTX)
P7	GBS	M&S	✓[b]	✓	×	×	No	✓	6	ND	×	×	1:800/ 1:800	×	✓ (red)	×	×	×	Death (Day 18)
P8	GBS	Motor only	✓[g]	✓	✓	✓	Nephrotic syndrome	× (deemed unsuitable)	6	ND[h]	×	×	1:400/ 1:400	×	✓ (red)	×	×	×	Death (Day 14)

Supplementary Table 2 - Clinical features of pan-neurofascin antibody positive patients

Key:
✓ (red) Given but no response
✓ (yellow) Given but only partial and/ or transient response
✓ (green) Given with good response
× Not given / not present / not done

[a] II-VII, IX-XII including optic disc swelling
[b] Facial diplegia, subtle gaze palsy
[c] Bilateral III, IV and VI nerve palsies, facial diplegia
[d] Mild bulbar weakness
[e] Facial diplegia, dysphonia
[f] Facial diplegia, complete opthalmoplegia, optic disc swelling with reduced visual acuity, bulbar dysfunction
[g] Facial diplegia
[h] Clonal urinary lambda light chain

ND: Not detected
CN: Cranial Nerve
CLL: Chronic Lymphocytic Leukaemia
MV: Mechanical ventilation
mRS: modified Rankin Score
CYC: Cyclophsphamide
FDB: Fludarabine
IVIg: Intravenous Immunoglobulin
PLEx: Plasma Exchange
ChlVPP: Combination chemotherapy for Hodgkins Lymphoma (Chlorambucil, Vinblastine, Procarbazine, Predinisolone)

Patient	Time from symptom onset	SNAPs	Motor Studies												EMG	Neurophysiologist's report
			Prolonged distal motor latency		Slowed conduction velocity			Abnormal F-waves			Conduction block		TD	CMAP		
			>110 %	>150 %	<90%	<85%	<70%	>120 %	>150 %	absent	>0.7	>0.5				
P1	2 weeks	Absent UL preserved sural												Globally absent	No signs of acute denervation	Inexcitable
	4 weeks	Globally absent												Globally absent	Positive sharp waves and fibrillations	Inexcitable
	8 weeks	Globally absent												Globally absent	Florid denervation with no recordable motor units	Inexcitable
P2	4 weeks	Marginal reductions in amplitude and velocity	1		3	3	1	2			3	2		<50% LLN x 1	ND	Demyelinating
P3	3 weeks [a]	Marginal reductions in amplitude and velocity	2		4	3				8	7	4		<80% LLN x1	Relative paucity of active denervation changes	Demyelinating
	6 weeks [a]	Absent UL SNAPs												Globally absent	Profuse acute denervation	Severe axonal loss
P4	4 weeks	Normal	4		2	2		2		6	4	4		<80% LLN x1		Conduction block
P5	Day 5	Mildly reduced UL amplitudes	3	2	2	1					1			<80% x6 <50% x 3	No spontaneous or voluntary activity	Demyelinating
	2 weeks		2											<80% x1 <50% x 1	Normal	Subtle demyelinating features, some improvement
	6 weeks	Absent / reduced	4	4	1	1	1	1	1	1				<20% x4 <10% x3	Pronounced active denervation	Secondary axonal loss
P6	3 weeks	Absent / reduced	3	2	2	2	1			1	2	1		<50% x5 <10% x4 NR x3	No spontaneous activity	Demyelinating
	5 weeks	Globally absent	1	1										<10% x4 NR x2	Profuse positive sharp waves and fibrillations	Severe axonal loss

Supplementary Table 3 - Detailed neurophysiological findings

Numbers refer to how many individual nerves met the criteria stated. SNAP – sensory nerve action potential, CMAP – compound muscle action potential, ND – not done. [a] Studies in this patient were performed 3 and 6 weeks after an acute neurological deterioration, which itself occurred after 4 months of insidiously progressive left leg weakness. Note the frequent presence of conduction block, absence of temporal dispersion, and frequent switch from a demyelinating/AIDP to axonal pattern on repeat testing, suggestive of nodal pathology ("nodopathy") [7].

Fehmi J, *et al. J Neurol Neurosurg Psychiatry* 2021; 92:1089–1095. doi: 10.1136/jnnp-2021-326343

	Treatment response (% of treated, n)															
	Steroids				IVIg				Plasma exchange				Rituximab			
**Antibody (n)**	**Total**	Good	Partial	None/ deteriorated	**Total**	Good	Partial	None/ deteriorated	**Total**	Good	Partial	None/ deteriorated	**Total**	Good	Partial	None/ deteriorated
**PanNF (8)**	**4**	0	0	0	**8**	0	25% (2)	75% (6)	**6**	0	50% (3)	50% (3)	**4**	100% (4)	0	0
**NF155 (17)**	**16**	19% (3)	37% (6)	44% (7)	**15**	6% (1)	27% (4)	67% (10)	**8**	0	25% (2)	75% (6)	**6**	50% (3)	33% (2)	17% (1)
**CNTN1 (11)**	**11**	36% (4)	27% (3)	36% (4)	**8**	0	50% (4)	50% (4)	**4**	0	0	100% (4)	**4**	50% (2)	25% (1)	25% (1)
**CNTN1/Caspr1 (9)**	**8**	13% (1)	50% (4)	37% (3)	**8**	0	13% (1)	87% (7)	**4**	0	50% (2)	50% (2)	**6**	67% (4)	33% (2)	0
**Seronegative (194)**	**62**	8% (5)	34% (21)	58% (36)	**83**	8% (7)	43% (36)	48% (40)	**27**	30% (8)	30% (8)	41% (11)	**1**	0	0	100% (1)

Supplementary Table 4 - Physician reported treatment response by serological group

IVIg – intravenous immunoglobulin. panNF – pan-neurofascin antibody positive, NF155 – neurofascin-155 antibody positive

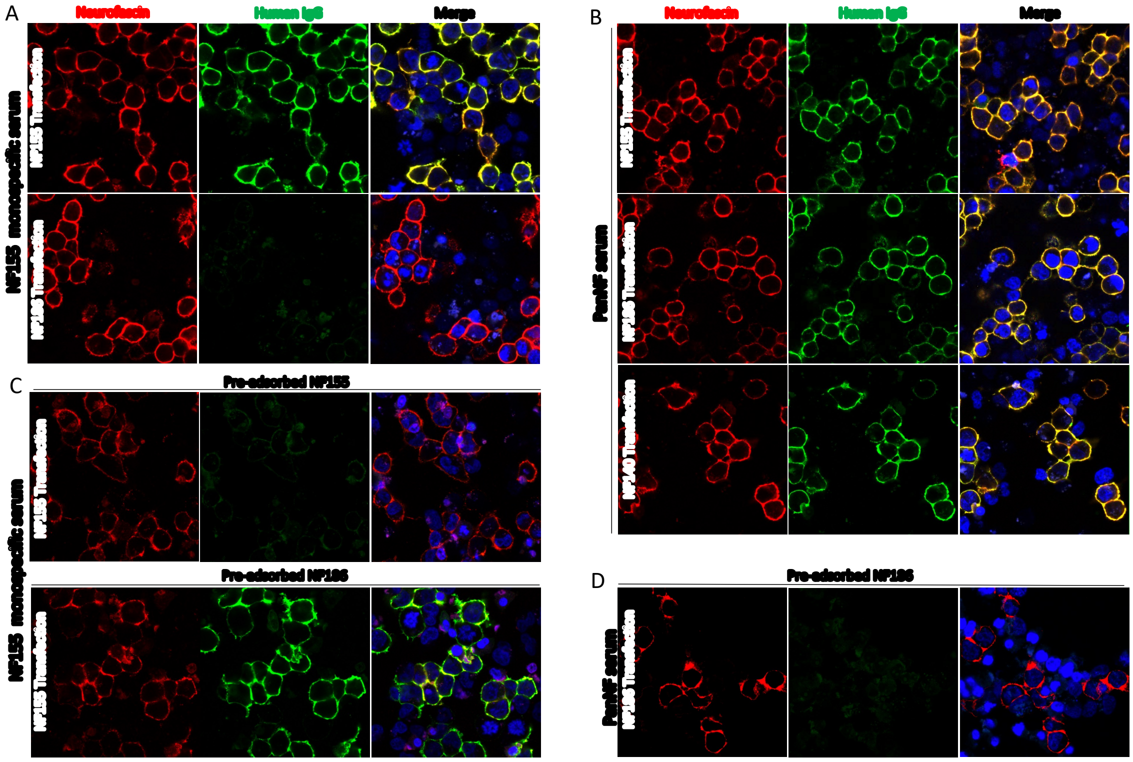

Supplementary Figure 1 - Comparison between neurofascin-155 monospecific and pan-neurofascin serum reactivity on cell-based assays

(A) Neurofascin (NF)-155 monospecific serum contains IgG (green) which reacts exclusively with the membrane of NF155 and not NF186 transfected cells (commercial neurofascin antibody, red). IgG4, IgG2 and IgG1 subclass antibodies are present in this example (bottom panel). (B) PanNF sera (patient 6) contains IgG reactive against NF155, NF186, and NF140 transfected cells. Reactivity to all three neurofascin isoforms was found in all patients. Only IgG1 subclass antibodies with this reactivity are detected (bottom panel).

Pre-adsorption of NF155 monospecific serum with NF155 protein, but not NF186 protein, abrogates IgG binding to NF155 transfected cells (C), in contrast to panNF sera, for which no IgG labelling to NF186 transfected cells can be seen after pre-adsorption with NF186 protein (D). Same results are obtained using NF155 transfected cells and pre-adsorption with NF155 protein.

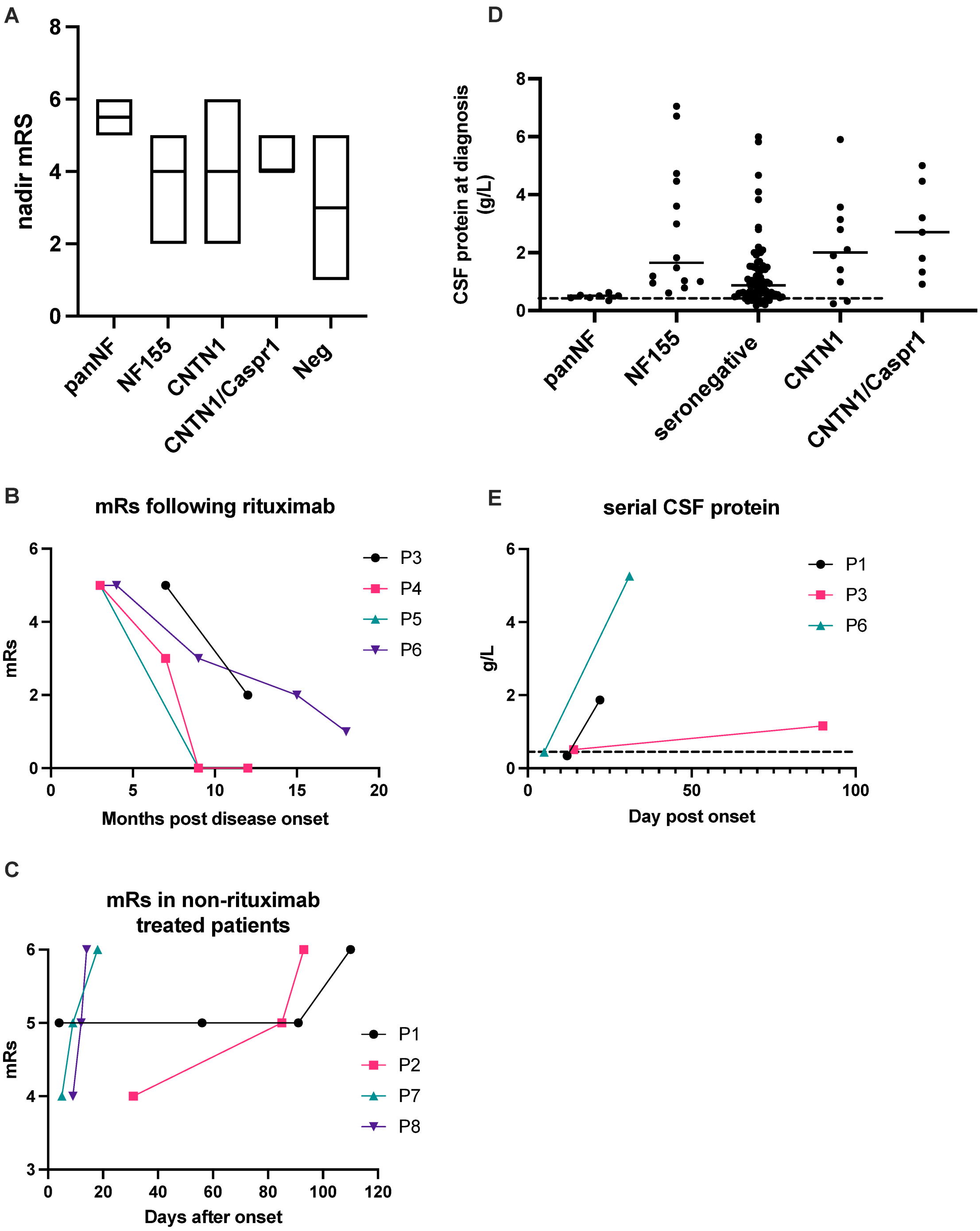

Supplementary Figure 1 - Modified Rankin scores and CSF protein levels

(A) The median nadir modified Rankin score (mRs) in the pan-neurofascin (panNF) antibody group was significantly higher than that in both the neurofascin-155 (NF155) antibody positive and seronegative groups. (B) Change in mRs in the 4 rituximab treated patients. The first datapoint for each patient represents the time of the first dose of rituximab. (C) Change in disability of non-rituximab treated patients. (D) CSF protein in the panNF group was either normal or only marginally elevated at diagnosis, and significantly lower than in both NF155 antibody positive and seronegative patients. (E) When performed, repeat CSF sampling showed abnormally increased protein levels at later time-points. Dashed lines indicate the upper limit of normal for CSF protein (0.45 g/L).

Fehmi J, *et al. J Neurol Neurosurg Psychiatry* 2021; 92:1089–1095. doi: 10.1136/jnnp-2021-326343

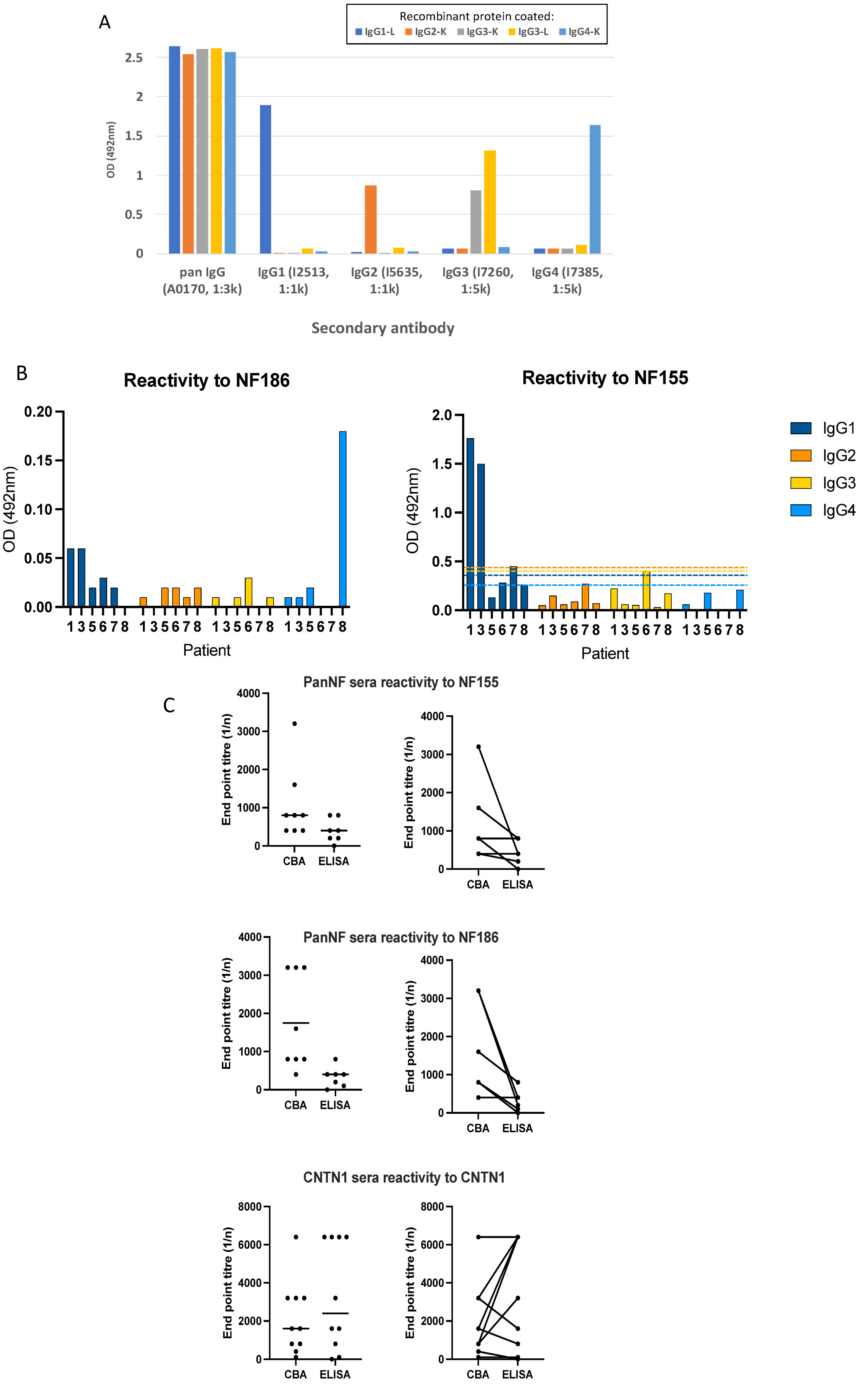
A
Recombinant protein coated:
IgG1-L IgG2-K IgG3-K IgG3-L IgG4-K
OD (492nm)
2.5
2
1.5
1
0.5
0
pan IgG (A0170, 1:3k)
IgG1 (I2513, 1:1k)
IgG2 (I5635, 1:1k)
IgG3 (I7260, 1:5k)
IgG4 (I7385, 1:5k)
Secondary antibody
B
Reactivity to NF186
Reactivity to NF155
OD (492nm)
0.20
0.15
0.10
0.05
0.00
2.0
1.5
1.0
0.5
0.0
1 3 5 6 7 8
Patient
IgG1
IgG2
IgG3
IgG4
C
PanNF sera reactivity to NF155
PanNF sera reactivity to NF186
CNTN1 sera reactivity to CNTN1
End point titre (1/n)
8000
6000
4000
3000
2000
1000
0
CBA
ELISA

12

Supplementary Figure 3 - Subclass specificity and comparison between CBA and ELISA

(A) A pan-IgG secondary antibody detects all 5 human IgG recombinant proteins similarly. The subclass antibodies produce a signal only in wells coated with recombinants of their specified subclass. (B) Sera from patients in this cohort are tested for antibody subclass reactivity to either NF186 or NF155. Cut-off optical density values for each subclass are indicated by the dotted lines (IgG1 0.35, IgG2 0.42, IgG3 0.40, IgG4 0.26), thus P1, 3 and 7 are positive for IgG1 antibodies only by ELISA. All other sera and subclass combinations result in OD results below the limits of detection. (C) End point titres on the cell-based assay (CBA) are consistently higher than by ELISA for both NF155 and NF186 antibodies but ELISA is at least equally as sensitive to the CBA for CNTN1 positive sera. The panels on the left-hand side show the individual data points and mean titre, the panels to the right show the same data with lines linking individual patients' results across the 2 assays.

Fehmi J, *et al. J Neurol Neurosurg Psychiatry* 2021; 92:1089–1095. doi: 10.1136/jnnp-2021-326343

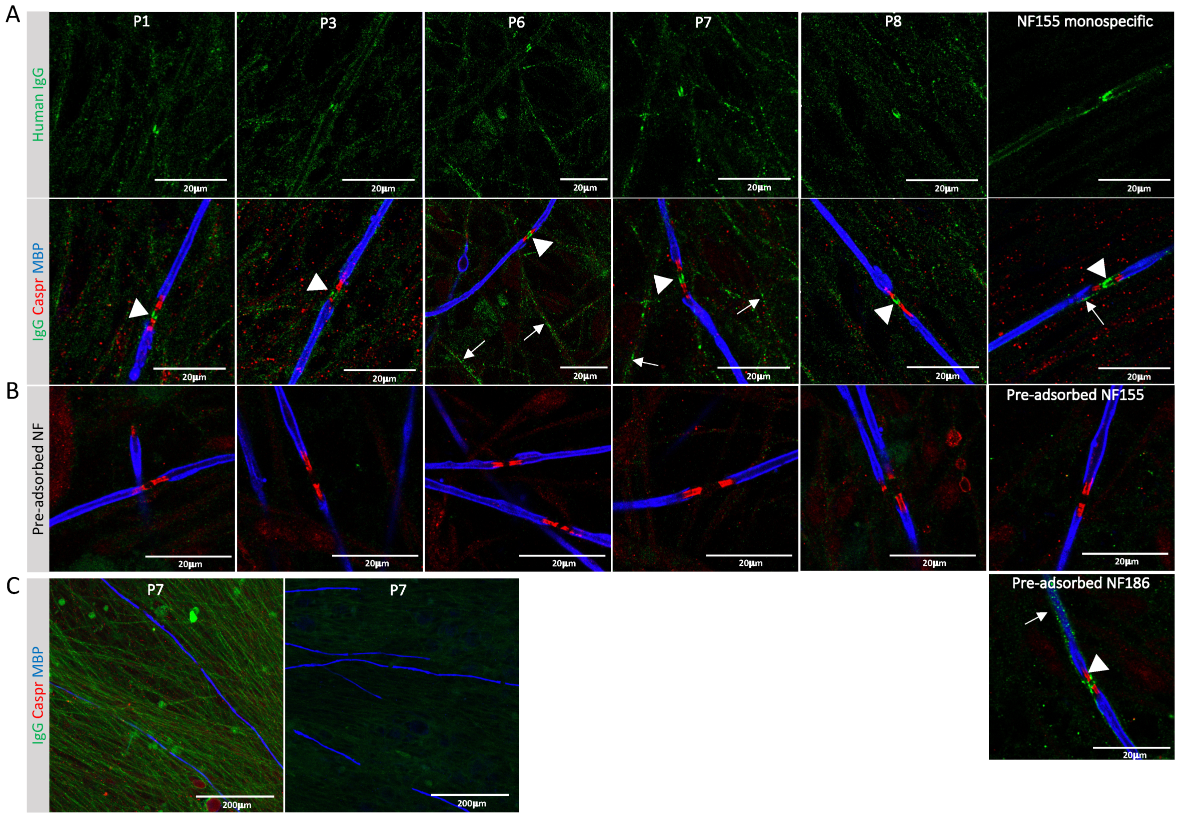

Supplementary Figure 4 – Pan-neurofascin serum is specific to NF155 and NF186 in live myelinating co-cultures

In myelinating co-cultures, high magnification confocal microscopy (x63 objective) reveals (A) IgG (green) is deposited at the node of Ranvier (arrowhead), flanked by paranodes labelled with Caspr (red), in patients 1, 3 and 8, with additional IgG labelling along axons (marked by arrow, axons not co-labelled in these images) in patients 6 and 7. There was no IgG binding observed for P2 and P5, and no sera was available for P4 for testing on cultures. Incubation of cultures with NF155 monospecific sera reveals IgG deposition over the outer surface of the myelin sheath (MBP, blue - arrow) and at the node. Nodal IgG staining was abrogated following pre-adsorption of panNF sera with either neurofascin 155 or 186 protein (pre-adsorption with NF186 shown in figure) (B). Axonal IgG labelling was also reduced following pre-adsorption in P6 and P7, best appreciated using lower magnification (x20 objective) (C). In contrast IgG labelling from NF155 monospecific sera is only abrogated when sera is pre-adsorbed with NF155, and is retained following pre-incubation with recombinant NF186 protein (far right column).

Fehmi J, *et al. J Neurol Neurosurg Psychiatry* 2021; 92:1089–1095. doi: 10.1136/jnnp-2021-326343

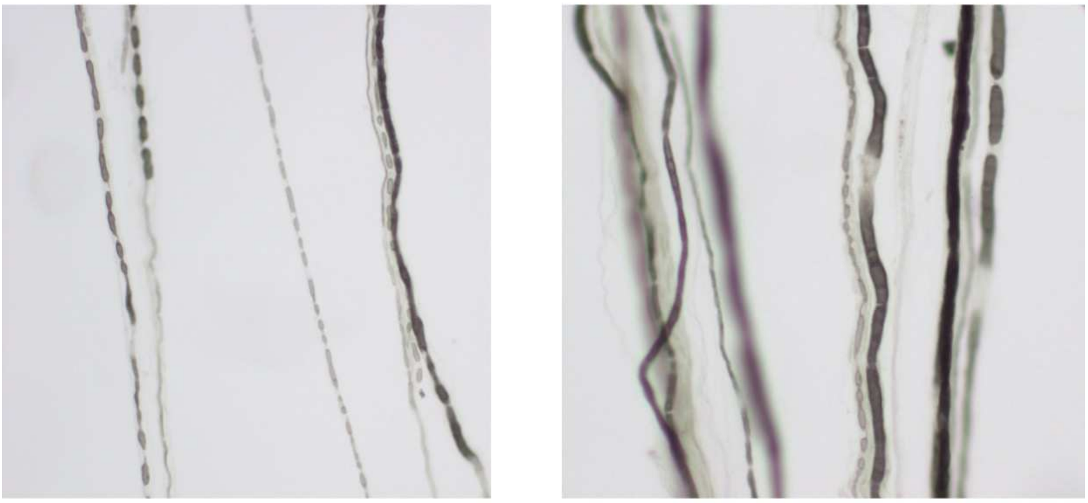

Supplementary Figure 5 - Teased nerve fibres

The Teased nerve fibres from the sural nerve biopsy of patient 6 show myelin ovoids consistent with axonal degeneration (left panel) and occasional short internodes (right panel).

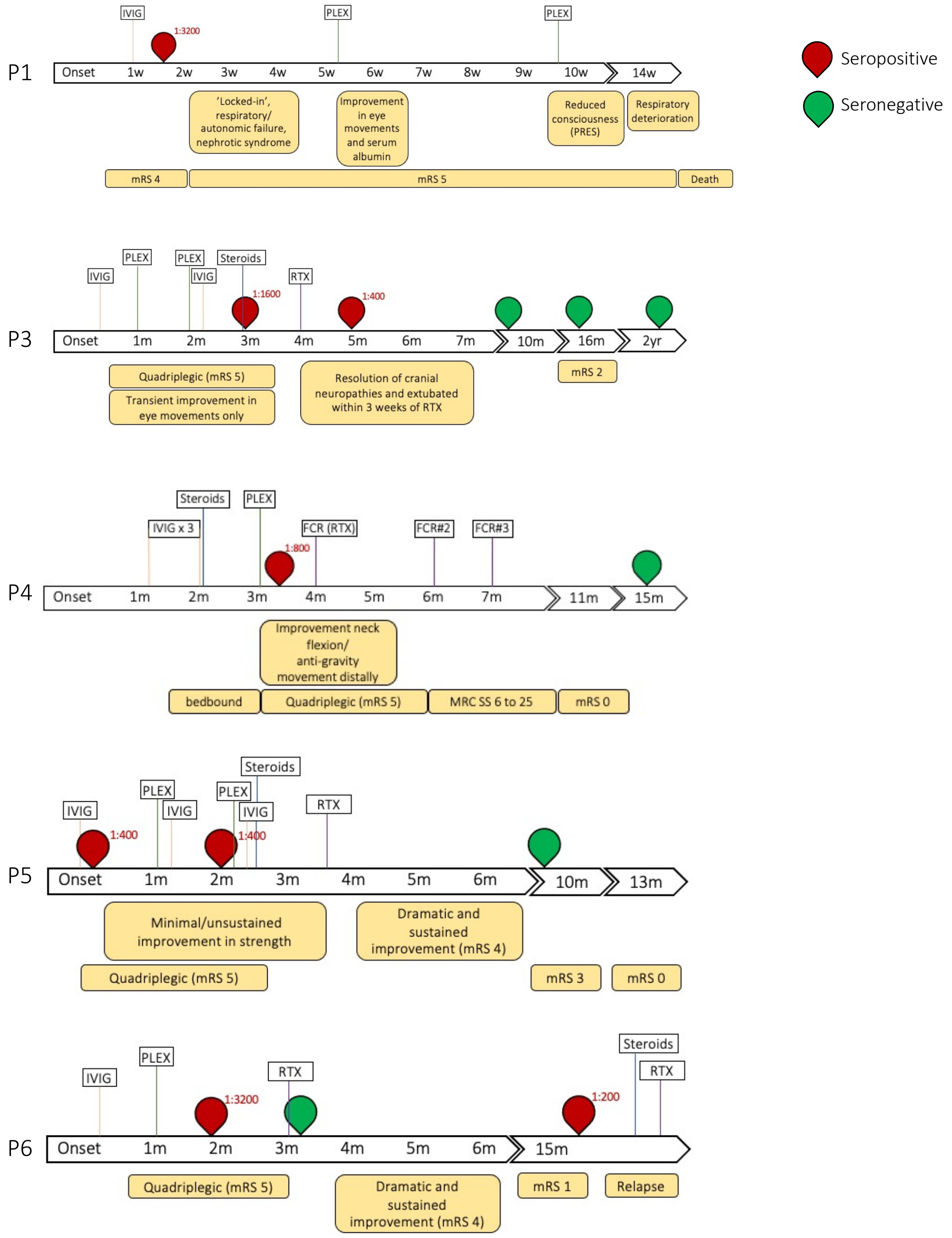

Supplementary Figure 6 – Treatment timelines

Treatments are indicated by flags at timepoints after onset lf symptoms (m – months, w – weeks). Functional status and recorded responses are noted in yellow boxes. Serological status, with antibody titre if positive, are indicated by red and green markers (see key in figure), and included where these were serially performed.
ChlVPP – Chlorambucil, Vinblastine, Procarbazine, Prednisolone (started for Hodgkins Lymphoma) FCR – Fludarabine, Cyclophosphamide and rituximab (started for CLL in absence of haematological indication as felt to be driving panNF antibody production), mRS – modified rankin score, PRES – Posterior Reversible Encephalopathy Syndrome

Case Vignettes

Patient 1

This woman in her 6th decade of life with a prior medical history of hypertension and Barret's oesophagus presented to her local hospital 4 days after developing distal upper and lower limb paraesthesia, and 1 day after first noticing bilateral leg weakness. On the day of her admission she had developed a severe, occipital headache. An initial CT brain scan showed sub-arachnoid haemorrhage, largely focussed over the left hemispheric convexity and particularly prominent in the left Sylvian fissure. She was transferred to the regional neurosciences centre the following day after further progression in her symptoms. By day 7 from the initial onset of symptoms she had proximal greater than distal weakness of all 4 limbs (and an MRC sum score of 30/60) with additional neck flexion weakness, but remained fully conscious with no cognitive deficits. She was now globally areflexic, with a normal sensory examination, and had a markedly labile blood pressure suggestive of autonomic dysfunction. At this stage she was bedridden but there was no evidence of cranial nerve involvement or neuromuscular respiratory failure. Further imaging showed no evidence of a cerebral aneurysm and no progression in the sub-arachnoid haemorrhage. A clinical diagnosis of GBS was made and she was commenced on intravenous immunoglobulin (IVIg, 2 g/kg given over 5 days). Unfortunately, she continued to progress. By day 10, her vital capacity, which had been normal (3.2L) on admission, had fallen to 0.69L, and she was intubated, ventilated, and transferred to ITU. Her serum albumin, which had been low normal (31 g/L) on admission, fell progressively to a nadir of 12 g/L by day 13. She developed worsening peripheral oedema and was found to have nephrotic range proteinuria. An opinion was sought from the nephrology team who advised against a renal biopsy. Her limb weakness progressively worsened, and by day 14 there was no detectable movement in any limb (MRC sum score 0/60). She was first noticed to have cranial nerve deficits on day 10, when bilateral lower-motor neuron facial weakness was observed. By day 14 she had complete paralysis of the entire cranial musculature with complete external and internal ophthalmoplegia. Throughout this time, she continued to demonstrate evidence of cardiovascular autonomic failure with an extremely labile blood pressure (systolic measurements ranging from 113 to 258) and episodes of tachy- and brady-cardia. Despite multiple anti-hypertensives, at some points including intravenous labetolol, her BP remained difficult to control with frequent hypertensive episodes. A repeat CT on day 14 demonstrated maturation of the SAH, with no other changes and no evidence of further bleeding or hydrocephalus. On day 16 she was noted to have bilateral swollen optic discs. A lumbar puncture on day 17 revealed an opening pressure >40cm CSF, with a CSF WCC of 2 and RCC of 1680. CSF protein was normal at this point (0.34g/L), although was later elevated at 1.87g/L on repeat testing on day 27. In view of the raised ICP and papilloedema, a lumbar drain was placed. Multiple further CT scans on days 18, 23, 25, 36 and 46 showed only progressive resolution of the SAH, with no hydrocephalus or evidence of developing intra-cranial pathology. Electrophysiological testing on day 18 showed that only sural responses were obtainable, and all other sensory and motor nerves were unresponsive. On day 24 the patient had a brief cardiac arrest when her tracheostomy tube temporarily blocked. Following 5 days of plasma exchange starting on day 40, she was able to move her eyes vertically to command and was able to use this movement to communicate. Her pupillary light responses also returned. On day 45 she experienced an episode of desaturation, bradycardia and brief (5s) asystole, in the context of a left, lower lobe pneumonia. Serial EEG recordings up to day 60 were either normal or showed findings consistent with mild sedation. On day 67, the patient was noted to be consistently less responsive, and was no longer able to move her eyes to communicate. Repeat CT imaging showed features suggestive of posterior reversible encephalopathy syndrome (PRES). Repeat EEG showed encephalopathic features. A further cycle of plasma exchange was started on day 69 but no clinical improvement was apparent. During both cycles of PLEx the serum albumin levels normalised but then quickly fell. When nerve conduction studies were repeated on day 88 all nerves were unresponsive. Repeat CSF analysis at this point was acellular and the CSF protein had normalised (0.258g/L). Further CT scans on day 89 and 96 showed improvement in the PRES changes, which were "barely visible" on the final images. There was an improvement in the background EEG between days 91 and 104, with better defined cortical rhythms, but no EEG response to verbal, tactile or noxious stimuli. Nephrotic range proteinuria (10g/L) persisted throughout this period. A further deterioration in respiratory function occurred on day 102, in the context of a further episode of pulmonary infection, with pseudomonas detected by broncho-alveolar lavage. On day 108, following discussion with the patient's family, respiratory support was withdrawn, and the patient died soon after.

Fehmi J, *et al. J Neurol Neurosurg Psychiatry* 2021; 92:1089–1095. doi: 10.1136/jnnp-2021-326343

Patient 5
This gentleman in his 7th decade of life presented 2 weeks after a flu-like illness with acute and progressive symmetrical predominantly distal sensory loss and weakness, areflexia, limb ataxia and profound sensory gait ataxia, rendering him immobile. He promptly developed facial diplegia, dysphonia, autonomic dysfunction (tachycardia with multiple premature ventricular contractions on ECG, diaphoresis) and respiratory failure requiring mechanical ventilation on the intensive care unit by day 2 of his admission. He had an iron deficiency anaemia (Hb 10.3 g/dL) with a normal OGD (unfit for colonoscopy), and was negative for anti-ganglioside antibodies, anti-MAG antibodies, paraneoplastic serology and autoimmune screen. A CT chest/abdomen/pelvis, CT head and unenhanced MRI whole spine, were unremarkable.

Despite making a prompt recovery after commencing IVIg, allowing extubation after 48 hours, he rapidly re-deteriorated and required further re-intubation within 4 weeks following a Klebsiella pneumonia. He had plasma exchange followed by IVIg at 1 and 2 months, followed by methylprednisolone and a tail of oral steroids with minimal, unsustained improvement in strength following each plasma exchange cycle. Finally, almost 3 months after admission to ITU he received rituximab and within weeks started to show dramatic and sustained improvement. By 6 months following rituximab he had regained independent mobility (modified Rankin score 3). Repeat panNF antibody testing at this point was negative.

Patient 6
A middle aged gentleman presented with progressive ascending bilateral symmetrical paraesthesia over a few days, followed by weakness in the same distribution, areflexia, and neuropathic pain. Other than a non-specific flu-like respiratory and gastrointestinal infection 6 weeks prior there was no more recent preceding illness. Objectively, all sensory modalities were affected, he had a severe sensory ataxia and developed quadriplegia with multiple cranial nerve abnormalities (II, III, IV, VI, VII, IX and X). He developed respiratory failure requiring ventilation on the intensive care unit. There was evidence of peripheral oedema with hypo-albuminaemia (19) although a urinary protein was not measured. Serology for HIV, Lyme, a paraprotein and anti-ganglioside antibodies were within the normal range and a CSF taken 4 days after admission revealed a normal protein and cell count. Nerve conduction studies initially performed 23 days after admission showed a diffuse, non-length dependent sensorimotor peripheral neuropathy with occasional conduction slowing and prolonged DMLs, possibly reflecting a demyelinating disorder. A subsequent study performed after another 15 days showed significant deterioration with no recordable sensory or motor potentials and features of severe axonal loss on needle EMG. An MRI of the brain was unremarkable, and lumbar roots only suggesting an S1 root impingement. The patient continued to deteriorate despite IVIg commenced 6 days into admission, and only sustained partial improvement with steroids, deteriorating further with PLEx at 1 month. Following treatment with rituximab, started 3 months after initial presentation, there was a clear and significant clinical improvement. PanNF antibodies were negative when retested at this point.

Fehmi J, *et al. J Neurol Neurosurg Psychiatry* 2021; 92:1089–1095. doi: 10.1136/jnnp-2021-326343

Online-only References

1 Delmont E, Manso C, Querol L, *et al.* Autoantibodies to nodal isoforms of neurofascin in chronic inflammatory demyelinating polyneuropathy. *Brain J Neurol* 2017;**140**:1851–8. doi:10.1093/brain/awx124

2 Fehmi J, Davies AJ, Antonelou M, *et al.* Contactin-1 Antibodies Link Autoimmune Neuropathies to Nephrotic Syndrome. Rochester, NY: : Social Science Research Network 2020. doi:10.2139/ssrn.3739819

3 Armbruster DA, Pry T. Limit of blank, limit of detection and limit of quantitation. *Clin Biochem Rev* 2008;**29 Suppl 1**:S49-52.

4 Clark AJ, Kaller MS, Galino J, *et al.* Co-cultures with stem cell-derived human sensory neurons reveal regulators of peripheral myelination. *Brain J Neurol* 2017;**140**:898–913. doi:10.1093/brain/awx012

5 Hadden RDM, Cornblath DR, Hughes R a. C, *et al.* Electrophysiological classification of guillain-barré syndrome: Clinical associations and outcome. *Ann Neurol* 1998;**44**:780–8. doi:10.1002/ana.410440512

6 Rajabally YA, Durand M-C, Mitchell J, *et al.* Electrophysiological diagnosis of Guillain–Barré syndrome subtype: could a single study suffice? *J Neurol Neurosurg Psychiatry* 2015;**86**:115–9. doi:10.1136/jnnp-2014-307815

7 Uncini A, Susuki K, Yuki N. Nodo-paranodopathy: beyond the demyelinating and axonal classification in anti-ganglioside antibody-mediated neuropathies. *Clin Neurophysiol Off J Int Fed Clin Neurophysiol* 2013;**124**:1928–34. doi:10.1016/j.clinph.2013.03.025
